# Capacitive heating devices for therapeutic hyperthermia: a review of essential physical characteristics and challenges

**DOI:** 10.1007/s00066-026-02540-1

**Published:** 2026-05-08

**Authors:** H. P. Kok, T. Ohguri, F. Navarro, U. S. Gaipl, C. A. Minnaar, J. Contreras, P. Ghadjar, J. Crezee, K. Kuroda

**Affiliations:** 1 Amsterdam University Medical Center, University of Amsterdam, Department of Radiation Oncology, Cancer Center Amsterdam, Meibergdreef 9, 1105 AZ Amsterdam, The Netherlands; 2 Department of Therapeutic Radiology, University Hospital of Occupational and Environmental Health, Kitakyushu, Japan; 3 Department of Medical Physics, Regional University Hospital of Málaga, Málaga, Spain; 4 Translational Radiobiology, Department of Radiation Oncology, Universitätsklinikum Erlangen, Friedrich-Alexander-Universität Erlangen-Nürnberg, Erlangen, Germany; 5 Department of Radiation Sciences, University of the Witwatersrand, 2193 Johannesburg, South Africa; 6 Department of Radiation Oncology, Hospital Regional Universitario, Málaga, Spain; 7 Department of Radiation Oncology, Charité—Universitätsmedizin Berlin corporate member of Free University Berlin and Humboldt University Berlin, Berlin, Germany; 8 German Cancer Consortium (DKTK), partner site Berlin, and German Cancer Research Center (DKFZ), Heidelberg, Germany; 9 School of Information Science and Technology, Tokai University, Tokai, Japan; 10 Center for Frontier Medical Engineering, Chiba University, Chiba, Japan

**Keywords:** Cancer, RF heating, Quality assurance, Phantom measurements, Treatment planning

## Abstract

Therapeutic hyperthermia is typically applied using radiative or capacitive heating devices. Capacitive heating applies electrode pairs positioned on the patient to increase the tumor temperature and is considered a user-friendly method for relatively easy application of hyperthermia to both superficial and deep-seated tumor sites. However, this heating technique also has some limitations that are mainly inherent to the physics of capacitively induced heating. This review provides an overview of the principles of capacitive heating devices and the factors influencing the power absorption and resulting temperature distribution in the patient. Device parameters that strongly influence the achieved temperature distribution include the electrode sizes, the water bolus temperature for skin cooling, the cooling medium, and the output power. These parameters vary in commercially available devices. Complete characterizations of most of the commercially available capacitive devices are still lacking. Sparse phantom measurements in the literature characterizing capacitive devices indicate that therapeutic heating is at least possible for superficial tumors and tumors at intermediate depth. The dominant E‑field orientation with capacitive heating induces preferential subcutaneous fat heating, which limits deep heating and makes it most effective for slender patients (i.e., fat layer thickness < 1.5–2 cm). Numerical simulations have been helpful in optimizing device design, particularly in terms of bolus cooling, and are increasingly used for patient-specific treatment planning. Based on the physical characteristics and present literature, it can be concluded that appropriate patient selection is important to ensure effective and responsible use of capacitive heating devices.

## Introduction

Hyperthermia is an effective sensitizing anti-cancer treatment modality that aims to increase tumor temperature within the therapeutic range of 39°–43 °C for 1 h [1]. In this temperature range, several temperature-related mechanisms become activated, such as inhibition of DNA damage repair mechanisms, increased blood perfusion, cytotoxic effects on hypoxic cells, and immune stimulation [2–10]. Activation of these mechanisms enhances the cytotoxic effects of radiotherapy and chemotherapy on tumor cells.

Several randomized trials have shown a significant improvement in local control and/or overall survival when hyperthermia is added to radiotherapy or chemotherapy—e.g., for cervical, rectal, bladder, head and neck cancers; recurrent breast cancer; sarcoma; and melanoma [11–16]—without significant enhancement of toxicity. Systematic reviews and meta-analyses indicated a ~ 20–25% increase in local control after adding hyperthermia to radiotherapy for patients with head and neck cancer, locally advanced cervical cancer, and locoregional recurrent breast cancer [17–19]. The clinical benefit of adding hyperthermia depends on the treatment quality. Multiple studies have reported associations between achieved thermal dose/temperature and clinical outcome [20–24]. Even though the strength of these correlations may vary by tumor site, the different analyses clearly indicate that clinical results improve with increasing thermal dose/temperature [20–24]. Hence, aiming for optimal temperatures in the therapeutic range should be a key quality objective in hyperthermia treatments.

Achieving optimal therapeutic temperatures requires high-quality heating equipment. Over the years, several types of radiofrequency (RF)/microwave heating devices have been developed, typically consisting of one or multiple antennas [25]. These devices vary in focusing ability, heating depth, and size of the focal heating zone. Consequently, dedicated devices for specific tumor locations are commercially available to ensure effective heating. For example, deep-seated pelvic tumors can be heated effectively using RF phased-array devices, such as the BSD 2000 systems (Pyrexar Medical, Salt Lake City, UT, USA) and the ALBA-4D device (Med-Logix Srl, Rome, Italy). These devices consist of an array of phase-amplitude controlled antennas positioned around the patient. The 60–120-MHz operating frequency ensures sufficient penetration depth and power control to achieve adequate tumor heating and avoid severe overheating of normal tissues. For superficial tumors extending up to 4 cm below the skin, good results can be achieved using superficial microwave antennas operating at 434 or 915 MHz (e.g., BSD 500 or ALBA ON 4000). Infrared heating using water-filtered infrared lamps (wIRA), e.g., using hydrosun-TWH1500 (Heckel medizintechnik GmbH, Esslingen, Germany), has shown good results for widespread superficial disease, infiltrating up to ~1 cm below the skin [26].

**Fig. 1:**
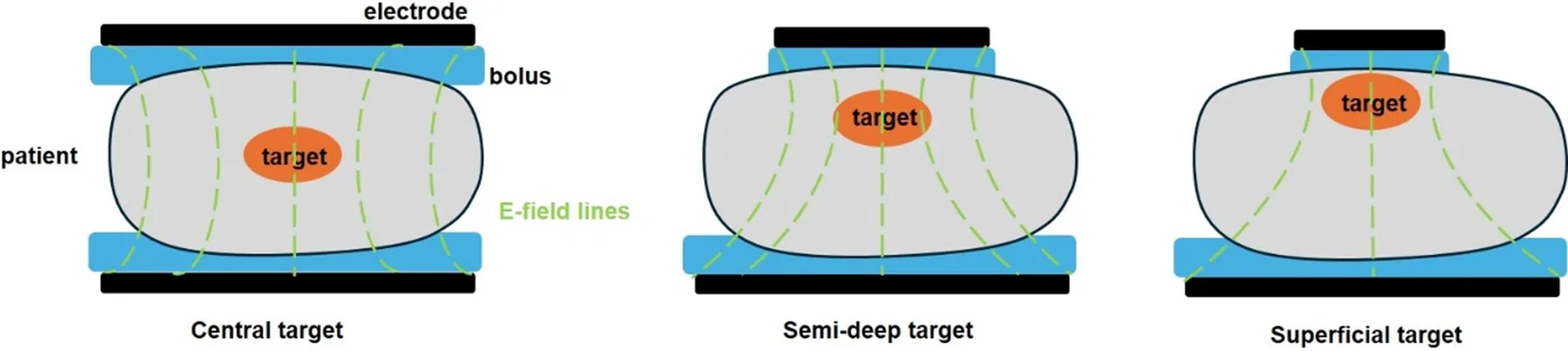
Schematic illustration of the influence of electrode size on heat deposition. Two equally sized electrodes are commonly applied to generate deep-seated heating. When a smaller electrode is used on one side, the field concentrates more near the smaller electrode; the penetration depth decreases and the heating becomes more superficial

In addition, capacitive heating devices have also been developed and are commonly used in oncological applications [25]. Capacitive heating is typically performed using two electrodes; an active electrode and a counter-electrode. Similar to RF/microwave heating devices, skin cooling by a water bolus is required. Therefore, the electrodes have an integrated water bolus bag. During treatment, the patient lies on a table with an embedded electrode. A second electrode is positioned on the opposite side on the patient. Consequently, a capacitive coupling with the human body is created and the resulting currents cause heating. When both electrodes have the same diameter, the absorbed power is concentrated at the center of the distance between the electrodes [27]. When the electrodes have different diameters, the power absorption concentrates near the smaller electrode, resulting in more superficial heating (see also Fig. 1; [27]).

Capacitive devices are thus considered a flexible and user-friendly method enabling relatively straightforward delivery of hyperthermia treatments for both superficial and deep-seated tumor sites. Capacitive hyperthermia devices are relatively affordable. Furthermore, room shielding with a Faraday cage is not required and treatments can be performed by a nurse, while the operation of RF/microwave devices requires well-trained dedicated staff. Especially in the case of deep hyperthermia, capacitive heating is also more comfortable for the patient, because of the more limited bolus pressure. However, despite these advantages, this heating technique also has some limitations that are mainly inherent to the physics of capacitively induced heating. When using capacitive heating in clinical hyperthermia applications, appropriate patient selection and effective use require awareness of the characteristics of the heating patterns generated by these devices. Therefore, the European Society of Hyperthermic Oncology (ESHO) Capacitive Hyperthermia Working Group^1^ initiated this review to provide a clear overview of the principles of capacitive heating devices and the factors influencing the power absorption and resulting temperature distribution in the patient. We discuss the physical principles of capacitive heating and the main differences between commercially available devices. Furthermore, we summarize the main results and insights from phantom measurements and numerical simulations reported in the literature characterizing this type of heating devices. We also discuss measures that have been suggested to mitigate the limitations of capacitive heating as well as the potential of treatment planning for capacitive hyperthermia treatments.

## Capacitive hyperthermia devices

Many capacitive heating devices are commercially available [25, 28], and examples of widely used capacitive systems are Thermotron RF8 (Yamamoto Vinita Co, Osaka, Japan), HY-DEEP 600 WM (Andromedic Srl, Velletri, Italy), REMISSION 1 °C (AdipoLABs, Republic of Korea), Oncotherm EHY 2000+ (Oncotherm Kft, Budapest, Hungary), Celsius TCS (Celsius42+ GmbH, Cologne, Germany), Synchrotherm (Synchrotherm, Vigevano, Italy) and the NRL-004 device (Jilin Maida Medical Equipment Co., Ltd., Jilin, China). The same basic physical principle of heating applies to all devices, i.e., heating is induced by currents resulting from a capacitive coupling of the electrodes with the human body. The most relevant device specifications are listed in Table 1.

**Table 1:** Examples of widely used commercially available capacitive heating devices with the main device characteristics. Output power refers to the maximum continuous net power. The last column indicates whether the device has been used in a randomized phase III study.

Device	Frequency	Bolus water	Output power	Electrode pairs	Electrode sizes	Phase III
*Celsius TCS*	13.56 MHz	Deionized	500 W	1	15, 20 cm	–
*Oncotherm EHY 2000+*	13.56 MHz	Deionized	150 W	1	10, 20, 30 cm	+
*REMISSION 1* *°C*^*a*^	0.46 MHz	None	120 W	1	3.2, 4.6, 6.6, 12, 15 cm	–
*Synchrotherm*	13.56 MHz	Deionized	600 W	1 (flexible)	12, 18, 26 cm	–
*Thermotron RF8*	8 MHz	Saline	1500 W	1	7, 10, 14, 21, 25, 30 cm	+
*HY-DEEP 600WM*	13.56 MHz	Deionized	600 W	1 (flexible)	17, 27 cm	–
*NRL-004*	30.32/40.68 MHz	Saline	1500 W	2	15, 20 cm	+

^a^The REMISSION 1 °C does not use bolus and the metallic electrodes are in direct electrical contact with the patient. Although this is technically a resistive heating device, because of the similarities in heating and practical use, this device is also included here

The main differences between the capacitive devices are the number of available electrode sizes (2–6) and their diameters (varying 3–30 cm), bolus filling (de-ionized water vs. saline, or no bolus), and maximum continuous net output power (ranging 120–1500 W; [27, 29, 30]). Operating frequencies vary between 0.46 and 40.68 MHz, but 8 and 13.56 MHz are most commonly used. Generally, in electromagnetic heating the wavelength, and thus the penetration depth in tissue, decreases with increasing frequency. For the relatively low frequencies applied in capacitive hyperthermia, the wavelength in muscle tissue is significantly larger than the distance between the electrodes. Consequently, the exposed tissue does not experience the full wavelength. This may explain why no clear optimal frequency can be identified from the literature. Most vendors of capacitive heating devices provide two or three different electrode sizes, typically in the range of 15–30 cm diameter. The Thermotron RF‑8 provides a larger variety of electrode sizes, ranging between 7 and 30 cm. The electrodes used by the Synchrotherm and HY-DEEP 600WM devices are flexible to adapt to the patient’s anatomy and improve electromagnetic coupling.

All devices use a single pair of electrodes, except for the NRL product line (Jilin Maida Medical Equipment Co., Ltd.), which uses two pairs of electrodes at slightly distinct frequencies. All electrodes are commonly circular, but these devices have additional rectangular (15 × 20 cm) electrodes placed on the left and right sides of the patient, parallel to the bed surface. The use of rectangular lateral electrodes is intended to provide better conformity with the patient’s body contour.

The HY-DEEP 600 WM device uses additional RF modulation protocols (Thermal Modulation System), with a pathology-specific noise modulation protocol. The Oncotherm EHY 2000+ also provides additional amplitude modulation. Here, the amplitude modulation starts at a given minimum modulation frequency f and then decreases with 1/f (pink noise) and a selected modulation index. It should be noted that amplitude/RF modulation is an added feature that has not been shown to have any effect on temperatures achieved [31], and temperature is, based on observed thermal dose–effect relations [20–24], still regarded as the major working principle of hyperthermia. The use of amplitude modulation aims to induce additional non-thermal effects in association with significant ion fluxes; the presence, potential mechanisms, and potential clinical relevance of these non-thermal effects remain the subject of ongoing research [32, 33].

## Capacitively induced heating

The heating patterns induced in the human body when using capacitive heating equipment are mainly determined by the electrode sizes, the number of electrode pairs, the anatomy of the individual patient and the thickness of the subcutaneous fat layer, the water bolus medium, and the bolus temperature. The influence of these factors is discussed in more detail in the next subsections.

### Electrode sizes

Selection of the electrode sizes is the main mode of power steering in capacitive hyperthermia, aiming to generate superficial, semi-deep, or more deep-seated heating. Two large (20–30 cm diameter) equally sized electrodes are commonly used for deep-seated targets, such as pelvic tumors. Most effective deep heating can be achieved when the electrode diameter is larger than the body diameter [34]. When the electrode diameter is larger than the thickness of the heating objects, spreading of current density is minimized, which improves the penetration depth [34].

More superficial tumor locations can be treated using a smaller top electrode, which results in a stronger power absorption near the smaller electrode [27, 35]. The larger the difference between the electrode diameters, the more superficial the heat deposition and the smaller the penetration depth, as illustrated in Fig. 1. The larger the range and number of electrode sizes, the better the flexibility considering heating depth. However, as can be seen in Table 1, most of the vendors typically provide two or three different electrode sizes.

### Electrode pairs

**Fig. 2:**
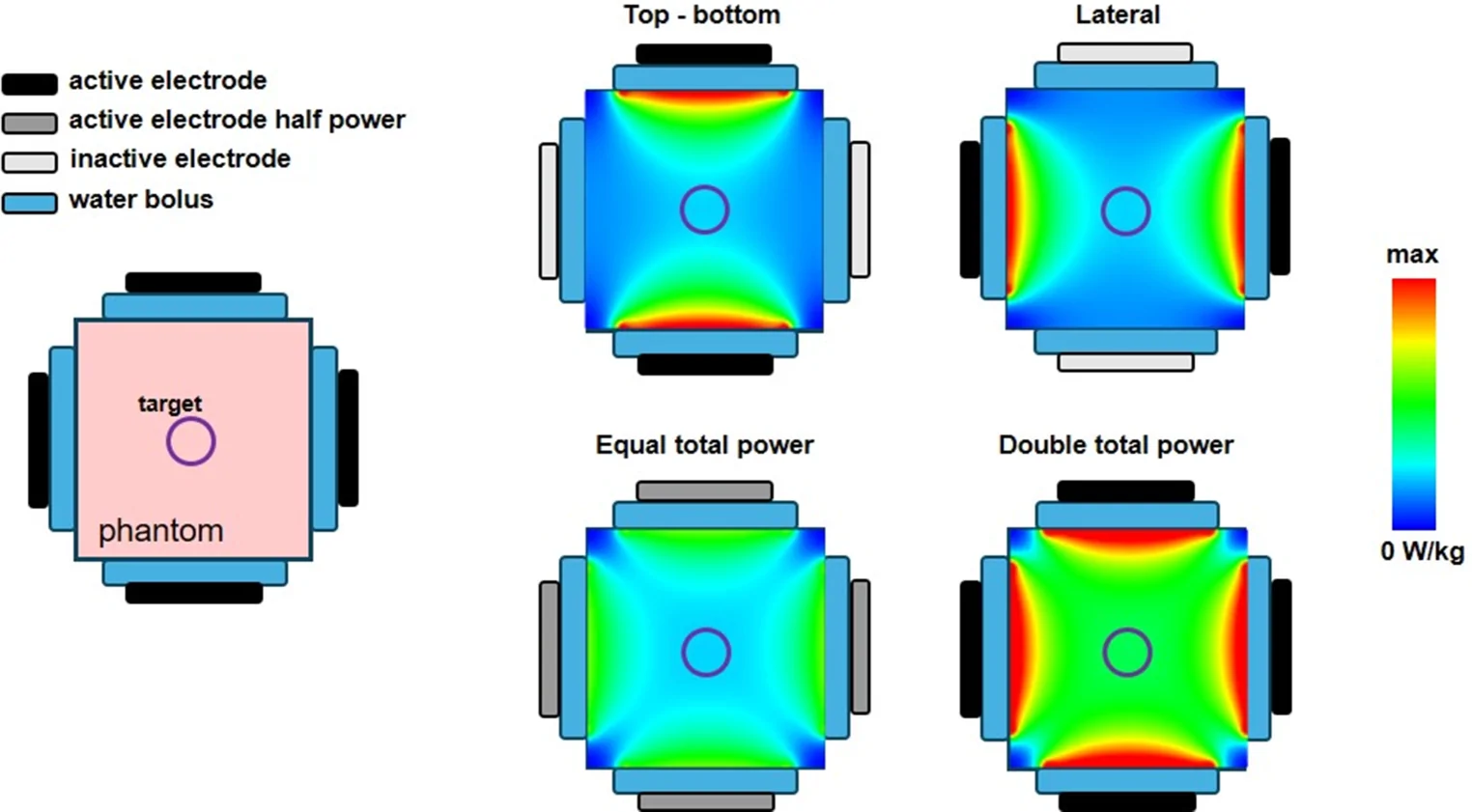
Illustration of a 4-electrode configuration and the effect on the absorbed power (SAR) distribution in a homogeneous phantom. By distributing the total power over two electrode pairs instead of one pair, the ratio between superficial and central power deposition becomes more favorable. This yields lower superficial SAR values and thus a lower risk of hot spots, while target coverage remains unchanged. This enables application of higher total power levels to achieve better target coverage, with maximum superficial SAR levels comparable to those for a 2-electrode configuration. NB: The figure is a schematic illustration only, and the exact SAR pattern achieved may depend on the voltage settings; no characterization of SAR patterns with a 4-electrode configuration was found in the literature

The use of a single electrode pair is most common for capacitive devices, but especially in China, devices are available with two pairs of electrodes, e.g., the NRL-004 device (Table 1). From a physics point of view, the advantage of using two pairs of electrodes is that, assuming the same total amount of power applied, the power applied per electrode pair is reduced by 50%. Consequently, by distributing the total power over two electrode pairs instead of one single pair, the ratio between superficial and central power deposition will become more favorable. This would also imply a relatively lower risk of superficial hot spots. In turn, this would enable the application of higher power levels to reach higher tumor temperatures. This is illustrated schematically in Fig. 2.

### Patient anatomy

Different tissues in the human body can differ considerably in dielectric properties (i.e., electrical conductivity and permittivity). In addition to the selected electrode sizes, these dielectric properties strongly determine the power absorption (or specific absorption rate; SAR [W/kg]) in the patient. Tissues with significant differences in dielectric properties are, for example, muscle/fat and muscle/bone [36]. The human anatomy typically includes a subcutaneous fat layer. The dominant electric field (E-field) produced by the capacitive electrodes is oriented perpendicular to the subcutaneous fat–muscle tissue interfaces (Fig. 1). According to Maxwell’s equations and the interface conditions, the tangential E‑field is continuous at the interface, while the normal E‑field component undergoes a discontinuity proportional to the permittivity of the two different tissues [37]:

1 $$E_{\mathrm{muscle}}^{| | }-E_{\mathrm{fat}}^{| | }=0$$

2 $$\varepsilon _{\mathrm{muscle}}E_{\mathrm{muscle}}^{\bot }-\varepsilon _{\mathrm{fat}}E_{\mathrm{fat}}^{\bot }=\sigma _{f}\mathrm{,}$$

with ε_muscle_ and ε_fat_ the (complex) permittivity of muscle and fat, respectively, and *σ*_*f*_ the free charge density at the boundary. Since the permittivity of fat tissue is much lower than for muscle tissue [36], the electric field in the subcutaneous fat layer becomes significantly higher. This explains the high risk of treatment-limiting hot spots at the fat–muscle interface during clinical applications of capacitive heating. The risk of severe hot spots is known to increase with increasing fat layer thickness [38]. It should be noted that this limitation by subcutaneous fat layers is typical for capacitive heating devices. Radiative devices largely mitigate this hot spot problem related to subcutaneous fat layers because these devices have the dominant field direction oriented along the patient axis [39]. Although radiative heating significantly reduces the risk, hot spots and treatment-limiting discomfort can still occur and are actively managed by adjustment of device settings [40, 41].

### Bolus medium

In addition to preferential heating of the subcutaneous fat tissue, electrode heating also induces surface hot spots around the margin of electrodes (edge effects; [34]). A water bolus, as used by most of the devices (Table 1), is meant to reduce these edge effects. Mitigation of edge effects is more effective when the bolus is somewhat larger than the electrode diameter [38]. An additional advantage of using a water bolus is that it provides a smooth transition to the body surface, thereby enabling a relatively homogeneous RF energy supply to irregular body surfaces.

There is some variation in bolus design between different capacitive devices. To avoid significant power loss by energy absorption in the water bolus, deionized water is often used, similar to radiative heating devices. However, the very low electrical conductivity of deionized water also yields a high electric resistivity, still resulting in a high local current density close to the electrode. When saline is used, the resistivity of the bolus is matched better to human tissues, yielding a smoother transition from electrode to tissue. This more effectively reduces the possibility of skin burns along the edges of the electrodes [42, 43]. Saline water as a bolus medium favorably affects the E‑field patterns inside the patient by improving the ratio of the central SAR with respect to the superficial SAR. Since the higher conductivity of saline results in a power absorption that starts inside the water bolus, significantly more output power is required to achieve therapeutic heating at depth. This explains why devices using saline boluses also need to have a much higher output power available (Table 1).

More effective suppression of the edge effects and thereby further improvement of the ratio of the central SAR to the superficial SAR maximum can be achieved with additional (electrolyte filled) overlay boluses, as used, for example, with the Thermotron RF8 [44, 45]. Overlay boluses cover a large part of the skin surface, thereby extending the surface area of the electrodes and resulting in diffusion of the electric current at the margins of the electrodes; this reduces the risk of hot spots [46].

### Bolus temperature

Bolus temperature is an important parameter in achieving an optimal balance between adequate tumor heating and minimal risk of excessive normal-tissue temperatures [47]. In the case of superficial tumors up to moderate depth, the bolus water temperature should be relatively high (~30–40 °C) to avoid undesired cooling of the treatment area.

For heating of deep-seated tumors, quite aggressive cooling is needed to mitigate high superficial temperatures caused by preferential heating of the subcutaneous fat [47]. Many devices typically use a cooling temperature between 10 and 20 °C when treating deep-seated tumors [48, 49]. A theoretical one-dimensional model of the bioheat equation predicted that excessive heating of a fat layer up to 1.6 cm thick can be avoided with effective bolus cooling at 10 °C [34]. To improve cooling in cases of thicker fat layers, an in-house-built external cooling unit was developed for the Thermotron RF8 that enables very aggressive cooling with an additional overlay bolus close to 0 °C [50]. Phantom experiments showed a significant temperature reduction up to ~2.5 cm depth [50]. Clinical results showed that this very aggressive cooling strategy enabled a ~ 50% power increase [50], resulting in 2–2.5 °C higher tumor temperatures for patients with colorectal cancer [47].

Most capacitive devices provide a flowing exchange of water in the bolus at a controlled temperature adequate for both deep and superficial applications (i.e., ~10–40 °C range). The Oncotherm EHY-2000 device does not provide temperature control, and cooling depends on the room temperature, which is typically 22–26 °C, thereby reducing controllability of superficial temperatures*. *The REMISSION 1 °C device does not use a cooling bolus, and the metallic electrodes are in direct electrical contact with the patient. Two of the available electrodes (3.2 and 4.6 cm diameter) feature integrated surface cooling using a Peltier element, which can be set at a 20 °C minimum.

## Feasibility of therapeutic heating: characterizing heating patterns

To gain insight into the heating characteristics of clinical heating devices, both phantom measurements and numerical simulations are very valuable instruments. Although phantoms are simplified and do not reflect the heating patterns in a human situation, these phantom measurements provide an important benchmark of heating characteristics of a device to determine whether it can generally provide sufficient temperature increase for therapeutic heating [51, 52], which is always an essential prerequisite for a clinical hyperthermia device. Numerical simulations can provide further insight into heating more realistic anatomies and perfused tissues. These instruments are therefore key to determine the general applicability for therapeutic heating of different tumor locations, i.e., deep-seated, semi-deep or superficial.

### Phantom measurements

To adequately determine the characteristics and heating abilities of capacitive devices for clinical use, important minimum criteria for phantom measurements are:

- Well-defined and reproducible experimental conditions, i.e., a phantom with representative and known dielectric properties
- Application of clinically representative power levels during the experiments
- Inclusion of multiple electrode size combinations in the measurement procedure to mimic different heating objectives (i.e., different heating depths)

To date, device characterizations meeting these criteria have been reported only for the Thermotron RF8 system and Celsius TCS devices [35, 53]. The next paragraphs briefly summarize the findings reported in these studies. Since the heating times in both studies were different, we converted the results reported in the publications to the effective temperature rise per min (°C/min), thereby enabling a more direct comparison and evaluation of the outcomes according to the ESHO quality assurance (QA) guidelines [52, 54].

#### Thermotron RF8:

**Fig. 3:**
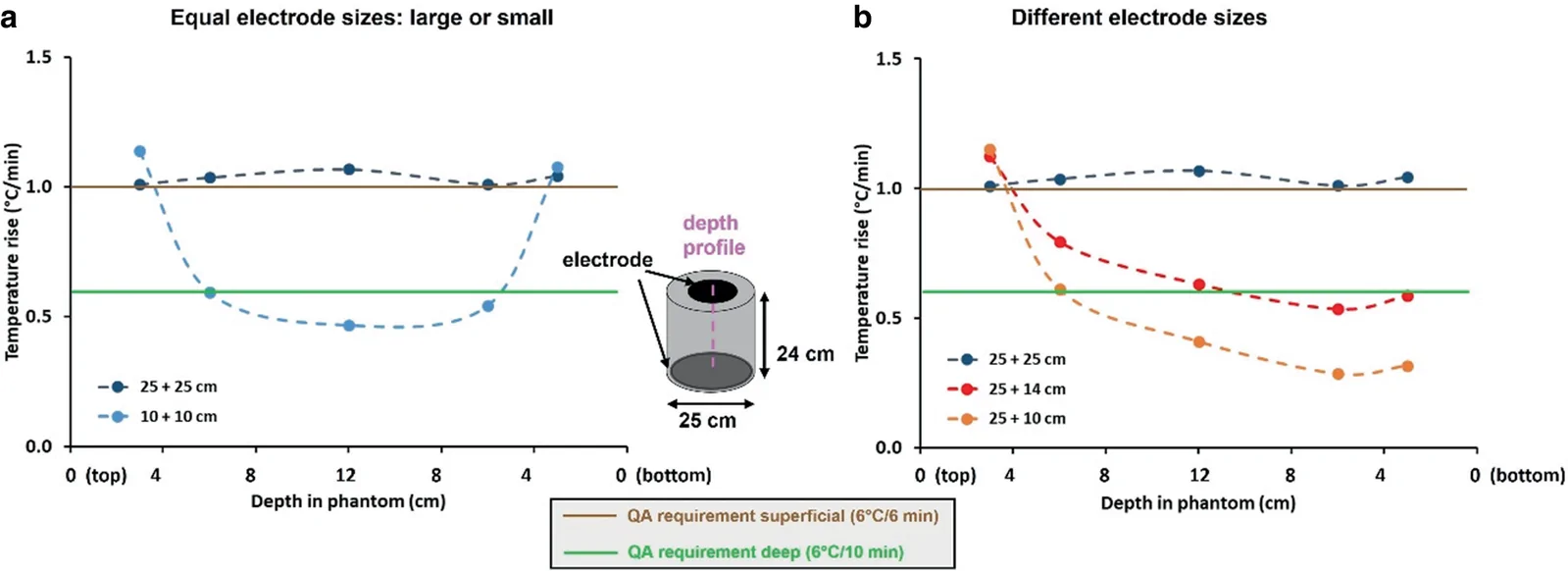
Depth profiles of temperature rises per minute using the Thermotron RF8 at 700 W, reconstructed from phantom measurements as reported by Song et al. [35], assuming an 18 °C baseline temperature. Deep-seated heating is more effective with equally sized, relatively large electrodes (**a**). It should be noted that the absence of a temperature gradient for the 25 + 25-cm electrode combination is explained by the fact that the diameter of the cylindrical phantom is also 25 cm. Therefore, the recent ESHO QA guidelines also prescribe that the lateral dimensions should be significantly larger than the electrodes [52]. Panel (**b**) shows that heating can be shifted toward the surface when using different electrode sizes. The horizontal lines indicate the minimum level of 0.6 °C/min and 1 °C/min for deep and superficial heating, respectively, as indicated by the ESHO QA guidelines [52, 54]

Song et al. performed measurements for the 8‑MHz Thermotron RF8 system at 700 W, using a cylindrical homogeneous agar phantom (4% agar gel; 0.2% NaCI; 25 cm diameter; [35]). Electrode combinations 25 + 25 cm, 25 + 21 cm, 25 + 14 cm, 25 + 10 cm, 10 + 10 cm were evaluated and water boluses were maintained at 20 °C. Figure 3 shows central depth profiles for different combinations of electrode sizes. In the case of two equally sized electrodes (Fig. 3a), larger electrodes clearly show a higher central temperature rise and thus a higher effectiveness for heating of more deep-seated tumor locations. When the top electrode is smaller than the bottom electrode (Fig. 3b), a more preferential superficial heating near the top electrode is observed, which increases with decreasing electrode diameter. These measurements demonstrate the possibility to control the depth of heating with the Thermotron RF8 by choosing properly sized electrodes [35].

#### Celsius TCS:

**Fig. 4:**
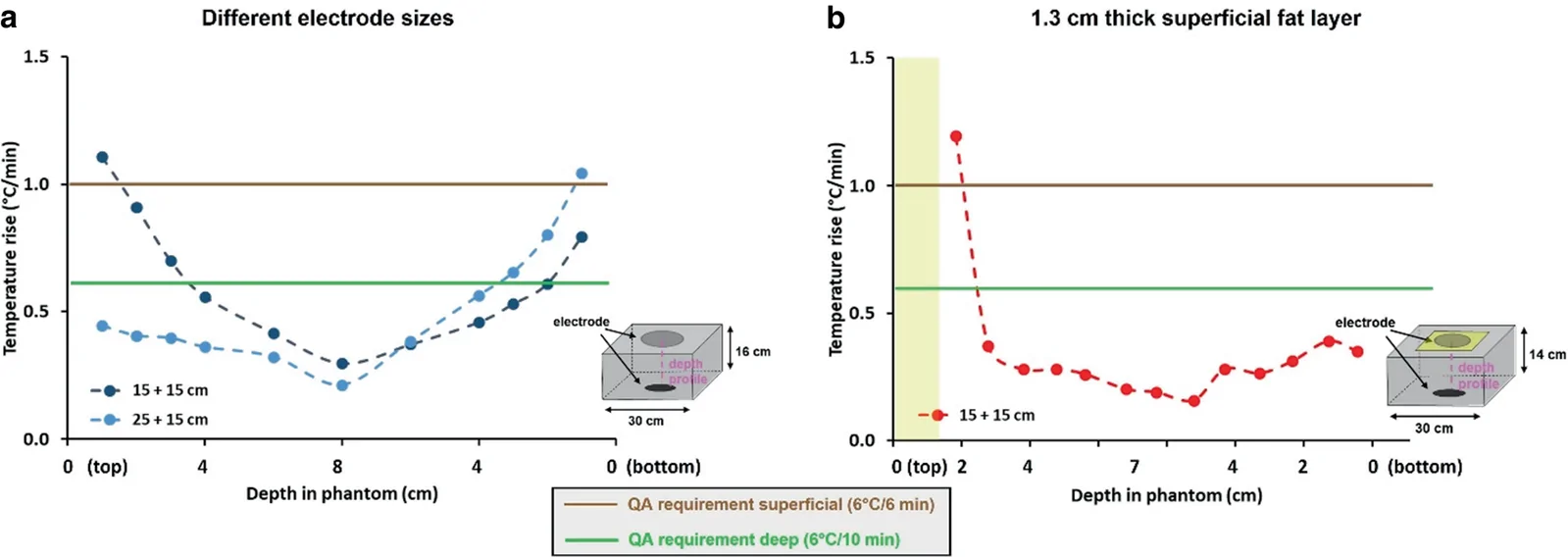
Depth profiles of temperature rises per minute using the Celsius TCS at 200 W, reconstructed from phantom measurements as reported by Beck et al. [53]. Panel (**a**) shows more superficial heating with the 25 + 15-cm combination, compared to two equally large electrodes. The slightly asymmetric profile for the 15 + 15-cm combination is explained by the interaction of the bottom electrode with the treatment table, which makes the bottom electrode effectively slightly larger [53]. Panel (**b**) shows the impact of a 1.3-cm-thick superficial fat layer. The fat layer absorbs a relatively large amount of the energy, yielding a significantly lower temperature rise in depth. The horizontal lines indicate the minimum level of 0.6 °C/min and 1 °C/min for deep and superficial heating, respectively, as indicated by the ESHO QA guidelines [52, 54]

Beck et al. thoroughly evaluated the temperature rise generated by the 13.56-MHz Celsius TCS device in homogeneous and inhomogeneous agarose phantoms [53]. The inhomogeneous phantoms had a 1.3-cm-thick superficial (porcine) fat layer embedded or an integrated bovine thigh bone. The power applied was 200 W and electrode combinations 15 + 15 cm and 25 + 15 cm were evaluated. Water boluses were at room temperature (~20 °C). Different electrode sizes again showed more preferential heating near the smaller electrode. The fat layer was shown to absorb a significant amount of power, thereby largely reducing the temperature rise levels beyond 2‑cm depth (Fig. 4b). Measurements in the agar–bone phantom showed a clear shielding effect of the bony structure, with very low temperature rises inside the cancellous bone [53].

#### Evaluation according to the ESHO QA guidelines:

As prescribed by the ESHO QA guidelines, effective therapeutic heating at depth requires a temperature rise of at least 6 °C in 10 min, i.e., 0.6 °C/min; for superficial heating, achieving 6 °C in 6 min is required (i.e., 1 °C/min; [52, 54]). These levels are indicated by the horizontal lines in Figs. 3 and 4. It should be noted that the absence of any temperature gradient for the 25 + 25-cm electrode combination in Fig. 3 is explained by the fact that the diameter of the cylindrical phantom is also 25 cm. The recent ESHO QA guidelines prescribe that the lateral dimensions should be significantly larger than the electrode sizes [52]. Therefore, no definitive conclusions regarding the feasibility of deep heating can be drawn from these measurements. For the Celsius TCS device, effective heating of deep-seated central tumors appears unfeasible. The phantom measurements for these capacitive devices thus indicate that therapeutic heating is at least possible for superficial tumors and tumors at intermediate depth, such as chest wall recurrences, breast tumors, or sarcoma in the limbs.

It should be noted that benchmarking phantom measurements do not account for tissue heterogeneities or temperature-dependent perfusion effects. Temperatures eventually achieved also largely depend on blood perfusion levels; for example, sarcomas are usually relatively easy to heat due to low perfusion levels in hypoxic tumor regions.

### Numerical simulations

Phantom measurements provide an initial basic and essential device characterization, as well as an indication of whether sufficient temperature increase can be achieved for therapeutic heating. However, as already mentioned earlier, the anatomy of human patients is much more heterogeneous, with considerable differences in dielectric properties and tissue perfusion. Therefore, numerical simulations can be a very valuable tool for obtaining additional insight into the expected effectiveness of heating a specific target location in realistic patient anatomies. Initially in-house-developed software was used for this purpose [55–59], but commercial packages supporting electromagnetic simulations (e.g., Sim4Life and COMSOL) are increasingly used [53, 60–62].

**Fig. 5:**
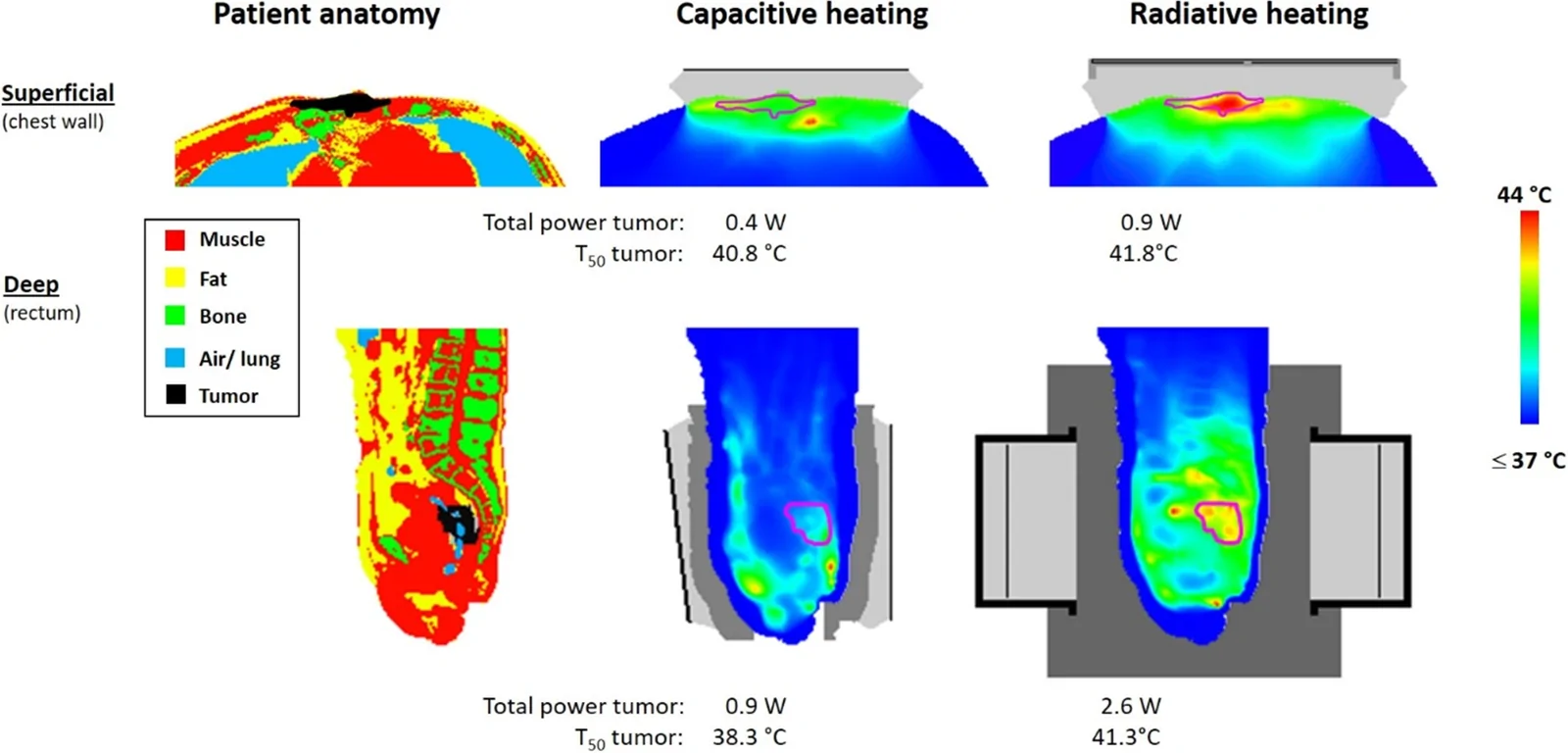
Numerical simulations can be applied to offer insights into the expected effectiveness of capacitive heating compared to radiative heating for different tumor locations. Feasibility of therapeutic heating with a capacitive device is predicted for a patient with a chest wall recurrence, while effective heating for the patient with a deep-seated rectal tumor is not predicted. Power levels were scaled such that the predicted maximum normal tissue temperatures reached 44 °C in each situation. Highest achievable power and temperature levels are predicted for radiative heating techniques. Simulations in this example were reproduced from open access references [39, 65]

Simulations in patient models have been used to evaluate the potential feasibility of heating specific tumor locations [55, 56, 63, 64], confirming the large risks of superficial hot spots and, consequently, the difficulties in achieving therapeutic heating at depth. Further investigations also compared the effectiveness of capacitive heating with radiative heating, both for superficial and deep-seated tumor locations [39, 64–66]. These simulations showed that capacitive heating is indeed capable of realizing therapeutic heating for superficial tumor locations, although (~0.5–1 °C) higher temperatures could be achieved with radiative equipment ([65]; Fig. 5). For deep-seated tumors, however, simulations indicate that capacitive heating can only achieve temperatures in the therapeutic range for very thin patients with almost no fat. Radiative heating was predicted to be overall more robust for all pelvic tumor sites evaluated (cervix, bladder, rectum, prostate), yielding target temperatures 1–3 °C higher than temperatures predicted for capacitive heating [39]. The difference in heating efficacy is explained by the aforementioned high-power absorption in the subcutaneous fat layers, although in practice, the exact difference will also depend on the specific device used, and especially the bolus medium that influences the power absorption in the fat layer [60]. Furthermore, in view of the uncertainties in tissue parameters used in simulations (see also Discussion section), these model predictions should be interpreted qualitatively. Radiative heating is thus generally predicted to achieve higher target temperatures, but the exact clinical benefit in individual patients may be different from the simulations. Although there are no clinical studies comparing radiative and capacitive heating, clinical observations have also shown that deep heating with capacitive devices becomes less effective with increasing fat layer thickness [23, 24, 38]. Ultimately, clinical feasibility of a heating modality can only be inferred from well-documented target temperature measurements and clinical outcomes.

## Numerical simulations to improve capacitive system design

Achieving therapeutic tumor temperatures is of utmost importance, considering the strong thermal dose–effect relationship observed in clinical hyperthermia [21, 23, 24, 67]. Since the high-power absorption in the subcutaneous fat layers is a recognized limiting factor in realizing optimal therapeutic temperatures in clinical capacitive hyperthermia, several studies have investigated adjustments to mitigate the preferential high superficial power absorption and improve therapeutic heating [60, 68]. Numerical simulations are very useful for investigating the impact of potential improvements in system design, since they provide the possibility to quickly evaluate the impact of different designs on identical anatomical models, compared to the original reference situation. The most important strategies evaluated are:

- Bolus optimization, i.e., optimizing the electrical conductivity
- Bolus-tissue contact optimization to avoid air gaps
- The use of mobile insulator sheets between the electrode bolus and the overlay bolus

### Bolus and bolus–tissue contact optimization

As indicated earlier in this review, the effectivity of bolus cooling is a crucial factor determining treatment quality for more deep-seated tumor locations, and the cooling effectiveness is determined both by the water temperature and the salinity. Earlier phantom studies and simulations with the Thermotron RF8 demonstrated a strong influence of the salinity of the overlay bolus on the SAR deposition in depth [45, 46]. Ohguri et al. performed simulations to further optimize the overlay bolus salinity and thereby the heating in more obese patients using the Thermotron RF8 [60]. Comparing simulations for NaCl concentrations of 0.1%, 0.5%, 1%, and 5% (5 or 10 °C) for a patient with cervical cancer showed that 0.5% NaCl at 5 °C resulted in the least overheated areas. Furthermore, an intergluteal cleft bolus insertion was proposed to avoid air gaps between the bolus and the patient, thereby further reducing the risk of hot spots and improving the temperature distribution. A similar strategy to optimize bolus–tissue contact using air-gap fillers was proposed earlier by Kumagae and Saito [69].

### Mobile insulator sheets influencing SAR patterns

Another strategy explored to improve deep heating with the Thermotron RF8 device is the use of mobile insulator sheets [68]. This is a simple method that does not involve any reconstruction of the heating device. Thin silicone rubber sheets were prepared in a circular sector shape to be used as insulator sheets to influence the shape of the generated SAR pattern. The sheets were positioned between the regular bolus and the overlay bolus and rotated manually during heating to influence the heating patterns. Simulations showed a clear improvement in the predicted SAR ratio between the subcutaneous fat and the target. Phantom measurements qualitatively confirmed the positive effects on the SAR distribution. A prospective clinical study was performed with 10 patients with pelvic tumors. Insulator sheets were used during the second and fourth treatment sessions, and CEM43T90 during these sessions was higher (median 1.9 min) than during the first, third, and fifth treatment sessions (median 1.0 min).

## Numerical simulations for treatment planning

A further step in the application of numerical simulations is to perform treatment planning to determine whether (capacitive) heating is feasible and to optimize treatment delivery. Special attention to feasibility should be paid in non-standard situations, such as when a patient has a metallic implant that could limit therapeutic heating or even impact treatment safety. Radiofrequency currents can concentrate around a metallic object, possibly yielding severe hot spots. Numerical simulation/treatment planning is very useful for evaluating such specific situations, for example, when the patient has an esophageal or biliary stent. Kato et al. performed extensive simulations with different stent orientations and blood flow rates [59]. Simulation results showed that the risk of hot spots is significant when the stent is perpendicular to the capacitive electrode, and increases further with increasing stent size. Based on these findings, they composed guidelines for the safe application of capacitive heating to patients with metallic implants [59].

Routine use of treatment planning for individual patients in clinical practice can help determine the best treatment strategy by comparing different treatment set-ups [70]. Using segmented patient computed tomography (CT) scans, patient-specific treatment planning can be applied to optimize treatment strategies for individual patients treated with capacitive hyperthermia, e.g., by selecting optimal electrode combinations and water bolus temperature and comparing prone and supine treatment positions [71]. The ratio between predicted target heating and the risk of hot spots can be used to determine the optimal choice for these treatment parameters for individual patients.

When using treatment planning to optimize treatment quality, validation of the accuracy and reliability of the simulations is of utmost importance. This requires well-defined phantom measurements, which serve as a benchmark. Beck et al. evaluated the reliability of predictions by Sim4Life using their phantom measurements for the Celsius TCS device [53]. Results showed that the general patterns are well predicted, but the simulations exhibited a significant overestimation of the SAR values at depth, up to a factor 2. As a result of this overestimation, the measured penetration depth of 4 cm in the homogeneous phantom was overestimated by 2–3 cm. This can have a significant impact on decisions based on planning predictions to optimize hyperthermia delivery. Similar results were obtained for predictions based on Sim4Life for the Oncotherm device. Muratoglu et al. showed a good qualitative match between measurements and simulations [61]. However, there were again large deviations when considering the absolute SAR: on average 25% (ranging from 7% to 36%) in a homogeneous phantom and on average 43% (ranging from 26% to 60%) in an inhomogeneous phantom with a superficial fat layer. Kok et al. validated planning predictions by Plan2Heat, for both the Thermotron RF8 device and the Celsius TCS device [71]. Measurement data were taken from earlier publications reporting phantom measurements for these devices [35, 53]. Simulations for all different homogeneous and inhomogeneous phantoms and electrode combinations showed a very good agreement with the reported measurements (within 5%). This large contrast in the performance of Plan2Heat compared to the other validation studies mentioned earlier is likely explained by differences in numerical solvers used in the different software packages and their suitability for capacitive simulations.

## Need for a specific quality assurance guideline for capacitive heating

Capacitive hyperthermia devices are popular since they offer advantages in accessibility, cost, and ease of implementation. However, from the topics discussed in this review it becomes clear that the physical characteristics and challenges observed for capacitive heating are substantially different compared to radiative heating techniques. Examples of important differences that have implications for clinical use are the following:

- The dominant E‑field direction for capacitive devices is perpendicular to the subcutaneous fat–muscle interface. This causes more preferential superficial fat heating compared to radiative devices, where the dominant field direction is parallel to the fat–muscle interface.
- The location of the heating focus with capacitive heating is determined by the electrode sizes, and consequently less spatially controllable, i.e., with fewer degrees of freedom, than with radiative devices using phase-amplitude steering.
- In the case of patient discomfort during treatment, radiative devices provide more direct measures to locally adjust the power deposition in the patient without reducing power in the target, e.g., by phase-amplitude adjustments. Options for similar direct measures are lacking with capacitive devices, where discomfort should be resolved by, e.g., total output power or bolus temperature adjustments.
- The operation of radiative devices is generally more complex, especially when using phased-array devices. This requires well-trained dedicated staff, while treatments with capacitive devices are less complex and often performed by a nurse.
- Radiative devices have been designed to either heat superficial lesions or more deep-seated tumors. Capacitive devices have been designed as a multi-functional system for both superficial and deep heating, depending on the electrode sizes selected for the specific situation. The effective heating depth depends on the electrode combination used. However, there is still no unambiguous answer to the question of whether capacitive devices are capable of effective therapeutic deep heating. Furthermore, there are substantial differences between different commercially available capacitive devices, especially in terms of available output power and electrode sizes. This will have an impact on the capability of heating specific (more deep-seated) target locations with different capacitive devices.

Consequently, clinical capacitive hyperthermia should be implemented under strict rules of quality assurance, clearly defining conditions for good hyperthermia treatment and identifying clinical conditions where a capacitive device can or cannot adequately heat the target region. The present ESHO QA guidelines largely focus on radiative heating techniques [51, 52]; therefore, a specific guideline is required for capacitive heating. Filling this gap will be an upcoming activity for the ESHO Capacitive Hyperthermia Working Group.

## Discussion

This review discussed the most relevant characteristics of capacitive heating for therapeutic hyperthermia. With capacitive heating, the achieved temperature distribution is mainly determined by the electrode sizes used, the water bolus temperature for skin cooling, the cooling medium, and the amount of output power. In addition to these device parameters, patient anatomy, and especially the thickness of the subcutaneous fat layer, largely determines the feasibility of therapeutic heating with a capacitive device, particularly for more deep-seated targets.

Increased subcutaneous fat thickness is consistently associated with reduced heating efficacy and increased risk of superficial overheating in capacitive hyperthermia. Several clinical studies have reported limitations in heating capabilities by capacitive heating in cases of increased subcutaneous fat layer thickness, which should ideally not exceed 1.5–2 cm to achieve therapeutic heating at depth with a capacitive device [38, 72, 73]. Hiraoka et al. reported thermometry results of capacitive hyperthermia applied with the Thermotron RF8 device for 60 deep-seated tumors at various anatomical locations (abdomen, pelvis, thorax, head and neck, or lower extremities; [38]). A clearly reduced heating efficacy was reported for patients with subcutaneous fat thickness measuring more than 15 mm. Furthermore, tumors in the head and neck, thorax, lower abdomen, and pelvis could be heated more effectively than tumors in the upper abdomen. Another study by Nakajima et al. included 21 patients with bladder cancer and measured bladder temperatures and subcutaneous fat temperature during treatment with the Thermotron RF8 [72]. Results showed a significant inverse correlation (*p* < 0.01) between subcutaneous fat thickness and bladder temperature, as well as a significant correlation between subcutaneous fat layer thickness and subcutaneous fat temperature. Therapeutic bladder heating to temperatures beyond 41 °C was possible for fat layer thickness below 2 cm. Ohguri et al. also reported a significant inverse correlation between subcutaneous fat layer thickness and tolerable output power for 35 patients with stage III non-small-cell lung cancer. Patients with fat layer thickness beyond 2.5 cm were excluded from hyperthermia. Even for these relatively thin superficial fat layers, median tolerable RF-output power ranged from ~550 W for a 2.5-cm fat layer up to ~1500 W when patients had almost no fat layer [73]. At these higher power levels, minimum intra-esophageal temperatures exceeded 41 °C. A retrospective analysis evaluating results obtained in patients with cervical cancer treated using the Thermotron RF8 showed that in about 45% of the patients (21/47), the minimum effective thermal dose (CEM43T90 ≥ 1 min) could not be achieved [23]. Significant efforts have been reported to optimize clinical application of the Thermotron RF8, including the use of overlay boluses, optimization of electrical conductivity within the bolus, improved contact at the gluteal cleft, and prone-position heating. These measures have enabled effective heating of patients with fat layers up to ~3 cm thick [60]. This resulted in achieving a CEM43T90 of 7.5 min (with a median of five total heating sessions) in patients with prostate cancer [24]. However, it should be noted that most devices use only electrode boluses with deionized water and relatively low power levels, and thus these optimized results cannot be generalized to common practice with capacitive devices. Overall, it can be concluded that when treating patients with deep-seated tumors and relatively thick fat layers, temperatures will likely remain at the lower end of the therapeutic range. This implies that, when using capacitive heating in clinical hyperthermia applications, appropriate patient selection is very important. This patient selection should take into account the achievable therapeutic temperature levels and the expected treatment effectiveness, thereby considering multiple factors such as tumor size, location, and depth, as well as the patient’s weight/BMI, physical condition, and disease progression.

As shown in Table 1, commercially available devices for capacitive hyperthermia mainly differ in the number and range of available electrode sizes, cooling medium, and maximum output power. Given the preferential subcutaneous fat heating with capacitive devices, effective deep heating strongly relies on the combination of sufficiently strong cooling and the availability of high output power. Devices using saline boluses and with high output power, e.g., the Thermotron RF8 and the NRL product line, therefore offer technical advantages for deep heating under current physical constraints, particularly in patients with thicker subcutaneous fat layers. This is also reflected by the fact that positive randomized clinical studies for deep-seated tumors treated with capacitive hyperthermia that improved complete response rates or overall survival were typically performed with these devices [30, 74, 75].

The feasibility of deep heating to therapeutic temperatures using relatively low power levels has not been established. A randomized study by Minnaar et al. in patients with cervical cancer using the EHY2000+ device (Oncotherm, Budapest, Hungary) reported significant benefits in 2‑year overall survival and in 2‑ and 3‑year disease-free survival rates [76]. However, interpretation of the results is complicated by multiple factors, among which is the absence of measured temperature data [77]. The study reported relatively low power levels (between 90 and 130 W) and the patients had a relatively high mean BMI (27.8 kg/m^2^; [78]). Thus, if therapeutic temperatures in the cervical tumor were achieved, these were probably near the low end of the therapeutic range. This is also further suggested by results from a partial characterization of the EHY-2030 device (Oncotherm; [61]). Temperature rise measurements in a homogeneous phantom with the 20-cm-diameter electrode at a 75‑W power level showed a maximum temperature rise of 3.6 °C in 6 min at 1‑cm depth [61]. The ESHO QA requirements of > 6 °C temperature rise could thus not be met at that relatively low power level, and at least 125 W would be needed to achieve a 6 °C temperature rise at 1‑cm depth. The aforementioned clinical study used the larger 30-cm-diameter electrode and up to 130 W [78]. For this situation, roughly half of the temperature rise achieved with the 20-cm electrode would be expected (i.e., ~3 °C), since the same amount of power is then distributed over a more than twofold larger body surface. Thus, since absence of measured temperature data, or very limited thermometry, significantly complicates interpretation of clinical results, adequate thermometry is of utmost importance, regardless of which heating device is used.

Tumor temperatures beyond 41 °C are required to induce DNA-repair inhibition by hyperthermia [2] and relatively low power levels are not likely to achieve such a substantial temperature elevation. The positive response in the clinical study by Minnaar et al. [76] might be attributed to other mechanisms, e.g., increased tumor oxygenation or drug delivery induced by enhanced blood flow as a result of mild heating. Effects such as perfusion and oxygenation are consistent with mild heating physiology. Lee et al. evaluated changes in temperature and blood flow in 20 patients with cervical cancer in response to heating with the Oncotherm EHY2000 device [79]. The temperature was measured using a temperature probe in the cervical ostium and tumor blood flow was measured using 3D color Doppler ultrasound. A significantly enhanced tumor blood perfusion was established with mean peri-tumor temperatures of 38.5 ± 0.8 °C [79]. Future research should reveal the relative contribution of different hyperthermia working mechanisms, including potential additional non-thermal effects and their clinical relevance [32, 33]. This could lead to mechanism-specific optimization across temperature ranges, provided robust thermometry is applied. However, although some beneficial effects might be observed in the lower end of the therapeutic range (i.e., around 39 °C), one should be aware that higher therapeutic temperatures will directly benefit clinical outcome, as supported by the significant thermal dose–effect relationships reported in the literature for both radiative [20–22] and capacitive devices [23, 24].

When numerical simulations are applied to optimize clinical treatment, validation of predictions in well-defined and reproducible phantom set-ups is important. Using such phantom measurements as a benchmark for validation also avoids uncertainties in dielectric properties. One of the most important parameters that should be considered in the validation is the penetration depth, as illustrated by the significant overestimation reported in some simulation studies [53, 61]. A large overestimation of the effective heating depth might influence conclusions based on numerical simulations.

In patient simulations, values for dielectric and thermal properties are required and these are usually taken from literature data, which have been conveniently summarized in specific databases, such as the IT’IS database (https://itis.swiss/virtual-population/tissue-properties/database), a database based on parametric models for dielectric properties developed by Gabriel (https://niremf.ifac.cnr.it/tissprop) and a database for thermal properties by McIntosh and Anderson (https://doi.org/10.1142/S1793048010001184). However, uncertainties in these values are unavoidable, given the large inter-patient variation in dielectric and thermal properties [36, 80]. Consequently, predictions of SAR/temperature levels achieved should not be interpreted as absolute, quantitative results. Adequate utilization of numerical simulations and treatment planning should qualitatively compare heating effectiveness in different situations, for example, situations with different electrode sizes or bolus temperature [70].

A relatively large number of capacitive devices is commercially available. However, compelling clinical evidence and a thorough characterization of the heating performance of these devices using multiple electrode sizes, clinically representative power levels, and phantoms with representative and known dielectric properties is limited to just a few of these devices. Future research should therefore focus on full characterization of the commercially available capacitive devices to determine their suitability for therapeutic hyperthermia at different depths according to specific QA guidelines. Furthermore, the availability of easy-to-use commercial software packages has led to an increased application of numerical simulations for (capacitive) hyperthermia. However, to ensure that simulations adequately represent the heating characteristics of a device, validation of SAR/temperature rise predictions against well-defined phantom measurements, serving as a benchmark, is essential. These commercial software packages use general numerical solvers and have been designed to allow their users to perform a wide variety of user-specified electromagnetic applications and model their own devices. Therefore, the user is responsible for validating each specific device implementation for hyperthermia applications in a well-defined benchmark set-up. Ideally, vendors of hyperthermia equipment should also offer validated and CE marked treatment planning packages for capacitive hyperthermia. To date, such planning packages are only available for radiative heating devices.

Clinical results achieved with capacitive hyperthermia indicate that capacitive equipment can be a useful part of the therapeutic armamentarium of clinical care [30, 74, 75]. However, when designing future (multicenter) hyperthermia trials, caution is warranted when intending to include both radiative and capacitive devices in the same study, unless quality assurance measurements have demonstrated effective heating capabilities of all devices for the specific target location. Generally, good quality assurance is essential to ensure effective application of capacitive heating and to avoid suboptimal treatments that might eventually damage the reputation of therapeutic hyperthermia as a very effective radio- and chemosensitizer. Furthermore, awareness of the basic principles and characteristics as described in this review might be helpful to clinicians in appropriate patient selection to ensure effective and responsible use of capacitive heating devices. The experience from clinical studies also emphasizes the clear need for active monitoring of tumor(-indicative) temperatures to ensure treatment quality [23, 30, 74, 75].

## Conclusion

The temperature distribution achieved in capacitive heating is largely determined by the electrode sizes used, the water bolus temperature for skin cooling, the cooling medium, and the amount of output power. These parameters vary in commercially available devices, thereby influencing the temperature levels that can be achieved with a device. In addition, the preferential heating of subcutaneous fat with this technique allows for the highest therapeutic temperatures to be achieved in slender patients (i.e., fat layer thickness < 1.5–2 cm). Therefore, appropriate patient selection is essential, thereby also accounting for the achievable therapeutic temperature levels and the expected treatment effectiveness. Characterizations of most of the commercially available capacitive devices, showing their suitability for therapeutic hyperthermia at different depths, are lacking; this should be the topic of further research.

## Data Availability

Data sharing is not applicable for this article; no new data were created or analyzed in this study.

## Footnotes

1 https://www.esho.info/esho-and-hyperthermia/esho/capacitive-hyperthermia-working-group/.
